# Comparative Analysis of Protein Glycosylation Pathways in Humans and the Fungal Pathogen *Candida albicans*

**DOI:** 10.1155/2014/267497

**Published:** 2014-07-03

**Authors:** Iván Martínez-Duncker, Diana F. Díaz-Jímenez, Héctor M. Mora-Montes

**Affiliations:** ^1^Laboratorio de Glicobiología Humana, Facultad de Ciencias, Universidad Autónoma del Estado de Morelos, Avenida Universidad 1001, Colonia Chamilpa, 62209 Cuernavaca, MOR, Mexico; ^2^Departamento de Ingeniería Genética, Centro de Investigaciones y Estudios Avanzados del IPN, 36821 Irapuato, GTO, Mexico; ^3^División de Ciencias Naturales y Exactas, Departamento de Biología, Campus Guanajuato, Universidad de Guanajuato, Noria Alta s/n, Col. Noria Alta, 36050 Guanajuato, GTO, Mexico

## Abstract

Protein glycosylation pathways are present in all kingdoms of life and are metabolic pathways found in all the life kingdoms. Despite sharing commonalities in their synthesis, glycans attached to glycoproteins have species-specific structures generated by the presence of different sets of enzymes and acceptor substrates in each organism. In this review, we present a comparative analysis of the main glycosylation pathways shared by humans and the fungal pathogen *Candida albicans*: *N*-linked glycosylation, *O*-linked mannosylation and glycosylphosphatidylinositol-anchorage. The knowledge of similarities and divergences between these metabolic pathways could help find new pharmacological targets for *C. albicans* infection.

## 1. Introduction

Although evolutionarily distant, humans and microorganisms of the* Candida* genus are closely related from a health perspective.* C. albicans* is a commensal organism that colonizes mucosal surfaces of the digestive tract and oral and vaginal cavities, and is able to cause superficial or systemic infections (candidiasis), particularly in the light of immunological host defects [1]. Nonetheless, other* Candida* species including* C. glabrata*,* C. krusei*,* C. parapsilosis* and* C. tropicalis* have also emerged as important causative agents of candidiasis. Intact glycosylation pathways in both, the human host and the fungal pathogen, are important, if not essential, for their development; thus, the knowledge of commonalities and divergences of these metabolic processes, as well as their functions, could help define pharmacological targets to suppress the pathogenicity of* Candida* and other fungal pathogens.

## 2. The* N*-Linked Glycosylation Pathway

The* N*-glycosylation pathway involves attachment of glycans to the amide nitrogen atom in the side chain of asparagine (Asn) residues of eukaryotic, archaeal, and bacterial glycoproteins. The best described model where the eukaryotic* N*-glycosylation pathway has been characterized in detail is the baker yeast* Saccharomyces cerevisiae* [2]. Through the years this model has helped to identify and characterize various human and fungal orthologs involved in this pathway.

**Figure 1:**
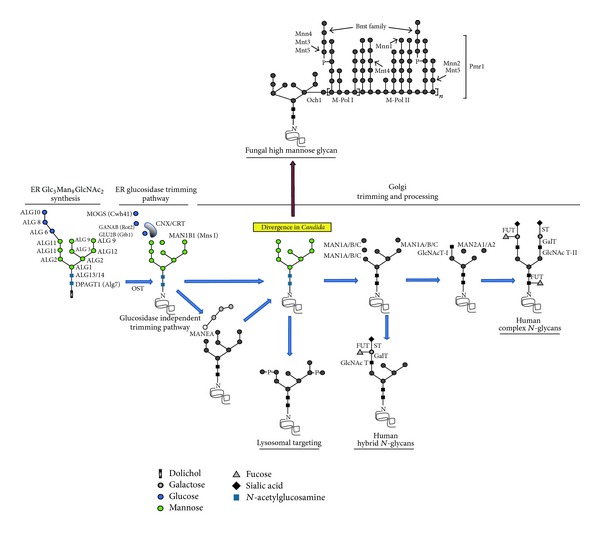
The* N*-glycosylation pathway. Commonalities and divergence in the* N*-linked glycosylation pathway. The shared structures between humans and* Candida albicans* have been colored, showing the rER synthesis of the Glc_3_Man_9_GlcNAc_2_ glycan and its transfer by the OST complex to a nascent protein. Once transferred, the Glc_3_Man_9_GlcNAc_2_ glycan is trimmed by the action of glucosidases and enters a quality control checkpoint performed by the CNX/CRT cycle. Once it passes this checkpoint, it is trimmed by mannosidase MAN1B1 to generate a Man_8_GlcNAc_2_ structure. At this point divergence occurs with* C. albicans* that synthesizes high-mannose glycans. In humans, the Man_8_GlcNAc_2_ structure is further demannosylated to Man_5_GlcNAc_2_ by Golgi mannosidases type I (MAN1A, MAN1B, and MAN1C). This* N*-linked glycan suffers further demannosylation and glycosylation processing by type II mannosidases (MAN2A1, MAN2A2), N-acetyl-galactosaminyl transferases (GlcNAcT), galactosyltransferases (GalT), fucosyltransferases (FUT) and sialyltransferases (STs). In humans, a glucosidase-independent trimming of Glc_3_Man_9_GlcNAc_2_ takes place, generating a Man_8_GlcNAc_2_ structure. In addition, lysosomal targeting of glycoproteins through modification with phosphate groups is only found in human cells.

The synthesis of the dolichol-linked glycan and its transfer to proteins are identical in both, human cells and* C. albicans* [3, 4] (see Table 1 and Figure 1). In fact, these processes are quite conserved among eukaryotic cells and there are only a handful of organisms where these stages are slightly different, such as trypanosomatids, some protists, and the fungal pathogen* Cryptococcus neoformans* [5, 6].

The eukaryotic* N*-linked glycosylation pathway is divided in two sequential stages: (a) synthesis in the rough endoplasmic reticulum (rER) of the dolichol-linked glycan precursor Dol-PP-Glc*N*Ac_2_Man_9_Glc_3_ and its transfer to a nascent protein and (b) the* N*-linked glycan processing and maturation in the rER and Golgi (Figure 1). Both stages require the action of different glycosyltransferases (GTs) and an adequate supply of donor substrates, which can be, depending on the GT family, nucleotide-activated sugars or dolichol-activated sugars.

The lipid dolichol used as a carrier in the first stage of* N*-glycosylation is a polymer of isoprene units (CH_3_–C(CH_3_)=CH–CH_2_–) predominantly of 14–17 units in the baker yeast [7], 19–22 in humans [8], and of undetermined length in* Candida*. Dolichol is modified in the rER cytosolic face by human and* C. albicans* orthologue GTs Alg7, Alg13/14, Alg1, Alg2, and Alg11, using the nucleotide sugars UDP-Glc*N*Ac and GDP-Man as donor substrates to synthesize the Dol-PP-Glc*N*Ac_2_Man_5_ intermediate. This intermediate is then flipped from the cytosol to the rER lumen, where synthesis proceeds by GTs Alg3, Alg9, Alg12, Alg6, Alg8, and Alg10 that use the dolichol-linked sugars Dol-PP-Glc and Dol-PP-Man as donors to synthesize the glycan precursor Dol-PP-Glc*N*Ac_2_Man_9_Glc_3_.

The flippase that translocates Dol-PP-Glc*N*Ac_2_Man_5_ has not been identified yet in any organism, although a critical accessory protein in yeast, Rft1, and its human ortholog have been proposed to participate in this process [9, 10]. Nonetheless, a recent work in* Trypanosoma brucei*, an early diverging eukaryote, has pointed that Rft1 is not the much sought after flippase, although it is critical for allowing maturation of the Dol-PP-GlcNAc_2_Man_5_ intermediate once it is flipped to the rER lumen [11]. A putative ortholog of Rtf1 has been also found in* C. albicans* (Table 1).

Once synthesized, the Dol-PP-Glc*N*Ac_2_Man_9_Glc_3_ precursor glycan is transferred* en bloc* by the oligosaccharyl transferase complex (OST) to Asn residues by linkage to carboxamide nitrogens. The Asn residues targeted for* N*-linked glycosylation are located, with rare exceptions, within the consensus sequence Asn-X-Ser/Thr (where X is any amino acid except proline) [12]. However, not all consensus sequences are* N*-linked glycosylated, because this protein modification is a co-translational process, and thus other factors are involved in selecting consensus sequences, such as accessibility of OST to the consensus sequence during the unfolded state of the protein. The OST complex has not been characterised in detail in* C. albicans*; however, the fungus encodes all the subunit orthologs found in* S. cerevisiae* OST, which is comprised of nine different transmembrane subunits: Wbp1, Swp1, Stt3, Ost1, Ost2, Ost3, Ost4, Ost5, and Ost6, where Stt3 is the catalytic subunit [13] (Table 1). Mammalian equivalents to yeast/*C. albicans* OST subunits are known and include: ribophorin I (Ost1) and II (Swp1), OST48 (Wbp1), defender against apoptotic cell death or DAD1 (Ost2), N33 (Ost3), magnesium transporter 1 (Ost6), and OST4 (Ost4) [14–16], (Table 1). In addition, two Stt3 protein orthologs (STT3A and STT3B) have been identified in plants, insects, and vertebrates [15, 17, 18]. The human STT3A isoform is primarily responsible for cotranslational modification of sequons when the nascent polypeptide enters the rER lumen. The STT3B isoform is less competent for cotranslational glycosylation, but mediates the posttranslational modification of skipped glycosylation sites in unfolded proteins [19]. The mammalian OST has been found in three complexes that exhibit different ribosome affinities and subunit compositions: OSTC(I),  OSTC(II), and OSTC(III) [16]. Furthermore, two additional components found in the mammalian OST complex have been reported: KCP2 and DC2 [16, 20].

Once transference onto the protein is achieved, the pathway continues with the processing and maturation stage. Processing is carried out, in both human and* C. albicans*, by rER enzymes: the mannosyl oligosaccharide glucosidase I (MOGS/Cwh41) that removes the outermost *α*1,2-glucose unit, and the mannosyl oligosaccharide glucosidase II which trims the following *α*1,3-glucose residue exposing the Glc_1_Man_9_Glc*N*Ac_2_ epitope [21] (Figure 1). In humans/*Candida,* glucosidase II is a heterodimer composed of two subunits, the hydrolytic*α*-subunit (GANAB/Rot2) and the *β*-subunit (GLU2B/Gtb1), see Table 1.

The Glc_1_Man_9_Glc*N*Ac_2_ epitope is a key point of ER quality control of glycoproteins, as it binds to the calnexin/calreticulin (CNX/CRT) lectin that is a folding sensor associated to ERp57. At this point, glucosidase II removes the last glucose residue and, if correctly folded, the glycoprotein exits the rER after the *α*1,2-mannosidase removes one Man residue from the middle branch of the* N*-linked glycan core, generating Glc*N*Ac_2_Man_8_ (Figure 1). If the protein is misfolded, the glycan is reglucosylated by the action of the UGGT1 glucosyltransferase in humans and its ortholog Kre5 in* C. albicans* [22]. UGGT is a conformational sensor, regenerating the acceptor substrate for the calnexin/calreticulin lectin, starting a new deglucosylation step by glucosidase II. This cycle continues until the protein is correctly folded or targeted for ER-associated degradation [23].

In contrast to* Candida*, humans code for an endomannosidase (MANEA) located in the Golgi/ERGIC compartment that provides a glucosidase I and II independent pathway for* N*-linked glycan maturation. MANEA is able to remove the inner most Glc residue along with the Man residue attached to it, generating the Glc*N*Ac_2_Man_8_ structure (Figure 1).

### 2.1. The Fate of Glc*N*Ac_2_Man_8_ in Humans

The further processing of the Glc*N*Ac_2_Man_8_ structure is the divergence point between humans and* C. albicans* (Figure 1). In humans, the* N*-linked glycans are processed by Golgi-resident mannosidase IA, IB, and IC, which have different hydrolytic patterns but all generate Man_5_GlcNAc_2._ This glycan is then acted upon by glycosyltransferase Glc*N*AcT-I to generate a GlcNAcMan_5_GlcNAc_2_ structure that is acted upon by type II *α*-mannosidases. The type II *α*-mannosidases include the Golgi mannosidase II (MAN2A1), and in some cell types, additional mannosidases MAN2A2 and mannosidase III have been described as bypassing enzymes when mannosidase II fails to hydrolyse the* N*-linked glycan core [24–26]. The type II *α*-mannosidases remove the terminal *α*1-3Man and *α*1-6Man residues allowing addition of a second GlcNAc residue to give way to complex glycans. The GlcNAc residues can be extended with additional monosaccharide linkages involving galactose, fucose, or sialic acid residues. Furthermore, the hybrid and complex* N*-linked glycans found in humans may exist with two or more GlcNAc-bearing branches or antennae. In forming multiantennary* N*-linked glycan structures, GlcNAc residues may be added to the trimannosyl core by six different GlcNAc transferases (I–VI) [27]. If type II *α*-mannosidases do not act or GlcNAcT-III bisects the GlcNAcMan_5_GlcNAc_2_ structure, hybrid glycans are then generated (Figure 1) [28].

In animals,* N*-linked glycans are terminated by sialic acid [29] in *α*2,3-, *α*2,6-, or *α*2,8- linkages by specific sialyltransferases [30]. In humans, sialic acid is mostly of the* N*-acetylneuraminic acid form, in contrast to most mammalian species, where a mixture of* N*-glycolylneuraminic acid and* N*-acetylneuraminic acid is generally found. Sialic acid can be further modified by acetylation or sulphation [31]. This monosaccharide in view of its terminal position, linkages, and negative charge has been an important element in the evolution of animal glycan function [32]. Although *α*2,3- and *α*2,6- sialic acid have been identified in the cell wall of* C. albicans* [33, 34], no ortholog to vertebrate sialyltransferase or ability to synthesize sialic acid has been characterized in this fungus [35]. However, evidence of sialic acid synthesis has been reported in* Aspergillus fumigatus* [36] and* C. neoformans*, where sialyltransferase activity has been identified [37].

Another frequent modification of human* N*-linked glycans not seen in* C. albicans* is *α*1,6- core fucosylation of the first Glc*N*Ac residue, as well as terminal fucosylation on Gal or Glc*N*Ac residues [38]. Nonetheless, fucose has been identified as a component of the cell wall of* C. albicans* [33] and binds the UEA-I lectin that is specific for L-fucose, more particularly to *α*1,2-fucose. UEA-I binding was associated with increased adherence to epithelial cells [39]. Recently mass spectrometry identified *α*1,6-fucose residues in oligomannosylated* N*-linked glycans of the fungi* Cantharellus cibarius* [40]. This raises the question on how this type of glycans are presented in the surface of mushrooms, as no* FUT8* family member of fucosyltransferases responsible for this linkage has been identified in yeast nor mushrooms [38]. Although little is known about fucosylation and sialylation mechanisms in* C. albicans* or fungi in general, more information is hinting at their role in pathological human host interactions through molecular mimicry.

Furthermore, human* N*-glycans can be phosphorylated to target glycoproteins to the lysosomes, through interaction with the Man-6-P receptor [41]. Phosphorylation occurs by modification of the Glc*N*Ac_2_Man_8_ structure by a UDP-Glc*N*Ac-dependent Glc*N*Ac-1-phosphotransferase (Figure 1) [42]. A Glc*N*Ac phosphodiester is added to* N*-linked glycans on one of three mannose residues on the arm with the *α*1,6- linkage to the core mannose. A second phosphodiester can then be added to the other side of the* N*-glycan or onto other mannose residues (Figure 1). Afterwards, the phosphodiester glycosidase in the* trans*-Golgi removes the Glc*N*Ac to generate Man-6-P residues. The phosphate residues partially block the action of processing mannosidases, maintaining the* N*-glycans in an oligomannosyl form. Some hybrid* N*-linked glycans with Man-6-P can also be found. This sorting system of soluble proteins does not exist in* C. albicans* or other yeast species, but interestingly they contain the gene* MRL1*, which seems to be the ortholog of that encoding the human Man-6-P receptor [43].

### 2.2. The Fate of Glc*N*Ac_2_Man_8_ in* C. albicans*

In* C. albicans*, the* N*-linked glycan Glc*N*Ac_2_Man_8_ is modified by proteins that have no human orthologs (see Table 2). The Glc*N*Ac_2_Man_8_ core is recognised by Och1, an *α*1,6-mannosyltransferase that adds the first mannose residue of the* N*-linked glycan outer chain [44] (Figure 1). This mannose residue works as a molecular primer to build the *α*1,6-mannose backbone, which in* S. cerevisiae* is elongated by the M-Pol I complex (a heterodimer composed of Mnn9 and Van1) that adds 3 to 7 mannose residues [45] and then by M-Pol II, a multimeric complex composed of Mnn9, Anp1, Mnn10, Mnn11, and Hoc1 [46, 47] (Figure 1). Both,* in vivo* and* in vitro* studies have shown that Mnn10 and Mnn11 contribute to most of the *α*1,6-mannosyltransferase activity of M-Pol II [47]. Thus far, there is only experimental evidence about Mnn9 role in* C. albicans* [48]; however, the encoding genes for all members of both complexes are present within* C. albicans* genome and it is likely they work as described in the baker yeast.

Parallel to this process, the *α*1,6-mannose polymer works as a molecular scaffold where branches of *α*1,2-mannose residues are added by Mnn5 [49, 50]. These are further elongated by the mannosyltransferases Mnt4 and Mnt5 [51], and members of the* MNN2*-like gene family [52]. In* S. cerevisiae*, the branches are terminated with *α*1,3-mannose residues added by action of the mannosyltransferase Mnn1 [53]. In* C. albicans*, these mannose residues are also present and are likely to be incorporated to glycans via the same protein [54]; however, it is most frequent that the *α*1,2-mannose branches are further decorated and capped with *β*1,2-mannose units [55]. The *β*1,2-mannosylation is characteristic of this pathogenic yeast species and is carried out by members of the* BMT* gene family [56].

Another decoration attached to the *α*1,2-mannose branches is the phosphomannan, which is a mannose residue attached to the* N*-linked glycan by a phosphodiester bond (Figure 1). This phosphorylation is not related to that found in humans and is partially synthesized by phosphomannosyltransferases Mnt3 and Mnt5 [51]. The identity of the enzymes involved in the addition of the rest of the phosphomannan remains unknown, although it is likely that members of the* MNN4*-like family contribute to this activity [57, 58]. As the* N*-linked glycan, the phosphomannan can be further *β*1,2-mannosylated with up to 14 *β*1,2-mannose units [59], by action of Bmt2, Bmt3, and Bmt4 [56].

### 2.3. Functions of* N*-Linked Glycans in Humans

The* N*-linked glycans associated to glycoproteins participate in the calnexin/calreticulin ER quality control system of glycoprotein folding [60]. Furthermore,* N*-linked glycans are involved in protein stabilization and trafficking and serve as moieties recognized by receptors, thereby modulating binding by increasing or decreasing affinity. The* N*-linked glycans play from trivial to essential roles in glycoprotein function and are involved in most, if not all, cellular processes. There is clear evidence that this posttranslational modification is essential for homeostasis in multicellular organisms as has been demonstrated by clinical phenotypes, mostly multisystemic, of patients affected by congenital disorders of glycosylation, indicating that* N*-linked glycan integrity is required for normal tissue function [61].

### 2.4. Functions in* C. albicans*

The* N*-linked mannans are essential for* C. albicans* viability, as demonstrated by treatment with tunicamycin, a drug that inhibits the action of Alg7 during* N*-linked glycan core synthesis [62]. Furthermore, they are quite important for cell fitness: defects in either the processing step by rER *α*-glycosidases or elongation by Golgi mannosyltransferases lead to longer duplication times, swollen cells, inability to perform proper cell separation, abnormal colony morphology, and impaired ability to undergo dimorphism [21, 44, 51, 63]. These pleiotropic defects are likely consequences of loss of the cell wall plasticity: mutant cells with defects in the* N*-linked mannan biosynthesis have rearrangements in the wall composition, including low mannan levels and high chitin and glucan contents, which led to increasing the sensitivity to cell wall perturbing agents such as tunicamycin, Congo red, Calcofluor white, hygromycin B, and caffeine [4, 21, 44, 50–52, 63]. Protein modification by* N*-linked mannans also modulates protein secretion, but surprisingly in a negative form, as shown in mutants lacking rER *α*-glycosidases, which display increased cell wall protein content [21]. In addition, biofilm formation seems to depend on* N*-linked mannans, as shown by the inability of tunicamycin-treated cells to form this kind of microbial consortiums [64]. Finally, and most important,* N*-linked mannosylation is required for normal cell adhesion and virulence [4, 21, 44, 50, 51, 63, 65–69]. The extent to which fucosylation and sialylation play a role in pathogenicity through the adhesion to the host surface, particularly extracellular matrix components, still requires further characterization in* C. albicans*.

## 3. The* O*-Linked Mannosylation Pathway

In contrast to* C. albicans* that only synthesizes* O*-linked mannosyl glycans (*O*-Man), six additional types of* O*-linked glycans are found in humans, and are classified based on the first sugar attached to the amino acid residue: GlcNAc, GalNAc, galactose, xylose, glucose, or fucose.

**Figure 2:**
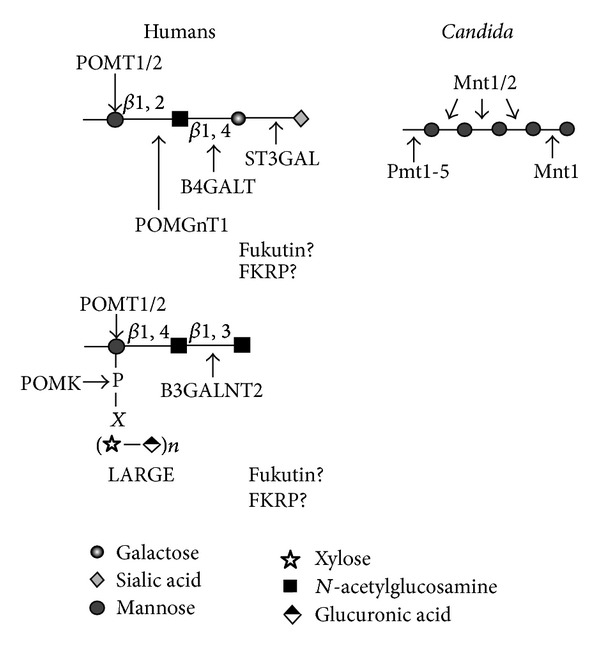
The* O*-linked mannosylation pathway. Human* O*-linked mannosyl glycans have been characterized mainly in alpha-dystroglycan where POMT1/2 add the first mannose residue that is further extended with other monosaccharides by the action of glycosyltransferases. The mannose residue can also be phosphorylated by the action of POMK, allowing further modification by disaccharide repeats of xylose and glucuronic acid synthesized by LARGE and LARGE2. Fukutin and FKRP genes are needed for correct glycosylation, but their roles remain to be clearly defined. In* C. albicans*, mannosylation involves the addition of the first mannose residue by the action of Pmt1-5, and that is further extended with four additional mannose residues by the action of Mnt 1 and Mnt2.

The* O*-Man glycans were identified on brain proteoglycans more than 30 years ago [70], and the* O*-mannosylation of *α*-dystroglycan (*α*DG) has been the most studied. In contrast to* C. albicans*, human* O*-Man glycans contain only one mannose residue (linked to the protein) and are extended with other monosaccharides (Figure 2). In* C. albicans*, the* O*-Man glycans are composed of up to five mannose residues [71]. Most of the mammalian* O*-mannosyl glycans are variations of the common tetrasaccharide core NeuAc*α*2-3Gal*β*1-4Glc*N*Ac*β*1-2Man*α*1-Ser/Thr, although branched structures with 2,6-di-substituted mannose (GlcNAc-linked *β*1,2 and *β*1,6) have been described in brain glycoproteins [72].

In humans, the first mannose residue is added in the rER by protein-*O-*mannosyl-transferase 1 (POMT1) [73] and 2 (POMT2) [74], homologous to* C. albicans* Pmt4 and Pmt2, respectively (Table 3). Both enzymes perform their function in an essential complex that uses Dol-P-Man as sugar donor [75]. In humans, elongation of* O*-Man glycans is initiated in the Golgi complex by transfer of Glc*N*Ac to the Man residue in the 2-OH position, mediated by the protein-*O-*mannosyl* N-*acetylglucosaminyltransferase 1 (POMGnT1) that uses UDP-Glc*N*Ac as donor substrate (Figure 2) [76]. Alternatively,* N-*acetylglucosaminyltransferase IX (GnT-IX) can make branched structures transferring Glc*N*Ac in *β*1,6-linkage to the* O*-Man glycan [22]. Further enzymes directly involved in the elongation of* O-*Man glycans remain to be identified among families of *β*1,4-galactosyltransferases and *α*2,3-sialyltransferases.

Another* O*-linked glycan structure (GalNac*β*1-3Glc*N*Ac*β*1-4Man*α*1-Ser/Thr) has been reported in *α*DG [77] (Figure 2). This structure is further phosphorylated in the 6-position of* O*-mannose by the action of the Protein-*O*-mannose kinase (POMK) [78]. The LARGE and LARGE2 bifunctional glycosyltransferases act on the phosphomannose structure producing repeating units of [-3-xylose-*α*1,3-glucuronic acid-*β*1-] (Figure 2) [79, 80]. Two other proteins, FUKUTIN and FKRLP, with glycosyltransferase characteristics, are also involved in the formation of human* O*-Man glycans, but their function remains unknown.

As in humans, in* C. albicans,* this pathway starts in the rER and finalises in the Golgi complex. The synthesis begins with the addition of one *α*-linked mannose residue to Ser or Thr residues via an ester bond. This reaction takes place in the rER lumen and is catalysed by the protein-mannosyl transferases that use Dol-P-man as sugar donor [81]. This enzyme activity is performed by a family composed of five members that are subclassified in three groups: the Pmt1 (Pmt1/5), Pmt2 (Pmt2/6), and Pmt4 subfamilies [82] (Figure 2). The proteins encoded by these subfamilies do not have redundant activity* in vivo*, as each member has specific substrates [81–83]. In addition, these enzymes interact among them generating protein-protein interactions. In* S. cerevisiae,* Pmt1 interacts* in vivo* with Pmt2, and combined disruption of* PMT1* and* PMT2* results in more than 90% less enzyme activity* in vitro* [84]. Another predominant complex includes Pmt5 and Pmt3, but in the absence of Pmt5, Pmt3 can form a complex with Pmt1, and Pmt2 can form a complex with Pmt5 when Pmt3 is disrupted [84]. Pmt1, Pmt5, and Pmt6 have no human orthologs (Table 2).

Once the glycoproteins are transported to the Golgi complex, the* O*-linked glycans are further elongated by the Golgi *α*1,2-mannosyltransferases Mnt1 and Mnt2 that have redundant activities to fully elongate the glycans [71, 85]. This mannan structure can also be phosphomannosylated, and in fact, the phosphosugar attached to* O*-linked mannans represents about 20% of total cell wall phosphomannan content [21]. However, the machinery involved in this process is different of that described for* N*-linked mannans, as Mnt3 and Mnt5 do not add phosphomannose to* O*-linked mannans [51]. Mnt proteins have no human orthologs (Table 2).

As mentioned before, sialic acid has been described in* C. albicans* [33, 36], and sialidase treatment has been shown to increase binding of the peanut agglutinin that has specificity for the Gal*β*1,3GalNAc sequence present in human Core 1* O*-glycans which has not been described in* C. albicans*. This suggests that sialic acid could be part of* C. albicans O*-linked mannans and the presence of uncharacterized galactosyltransferases.

### 3.1. Functions in Humans

The best characterized mammalian* O*-linked Man glycoprotein is *α*DG (Figure 2). This protein is a glycosylated peripheral membrane protein involved in linking the cytoskeleton of neurons and muscle cells to the basal lamina through interactions with extracellular proteins; glycosylation of *α*DG is essential for its function [86]. To date, mutations in seven glycosyltransferase or glycosyltransferase-like genes have been reported to affect the* O*-linked mannosylation pathway and are causative for various forms of autosomal recessive congenital muscular dystrophies associated with variable brain and ocular abnormalities [87]. As it was mentioned earlier,* O*-Man glycans in humans are not highly mannosylated structures; they only possess a single Man residue. This divergence is functionally important in the immune systems recognition of pathogenic yeast and fungal microorganisms, including* C. albicans*. Highly mannosylated structures, as those found in yeast and fungi, are recognized as foreign by both circulating antibodies and elements of the complement system, including both the classical and alternative pathways [88].

### 3.2. Functions in* C. albicans*

Loss of* PMT2* or combined disruption of* PMT1* and* PMT4* led to nonviable cells, indicating that* O*-linked mannosylation is essential for growth and cell viability [81]. In addition, incomplete* O*-linked mannan elaboration has been associated with rearrangements in the cell wall composition, increasing sensitivity to cell wall perturbing agents, defects in morphogenesis, reduced tissue adhesion, defective biofilm formation, and virulence attenuation [71, 81, 89–91].

The* O*-linked mannans are key cell wall elements during the* C. albicans* sensing by immune cells. This cell wall component is sensed by TLR4 receptor [88], and loss of either* O*-linked mannans or TLR4 receptor has a negative impact on cytokine production by human PBMCs [88], on the proinflammatory response of oral epithelial cells [92], and on yeast killing by human polymorphonuclear cells [93]. Indeed, TLR4^−/−^ knockout mice are more susceptible to infections caused by* C. albicans* due a defective immune response against the fungus [94, 95]. Furthermore, it has been demonstrated that simultaneous stimulation of dectin-1 and either TLR2 or TLR4 significantly enhances cytokine production in both human monocytes and macrophages [96, 97]. Therefore, it has been hypothesized that recognition of* O*-linked mannans plays a pivotal role, along with *β*1,3-glucan sensing, in the establishment of a protective anti-*Candida* immune response.

However, the relevance of* O*-linked mannans during* C. albicans* sensing is not the same for different kinds of immune cells, as yeast cells lacking both* MNT1* and* MNT2*, and therefore expressing truncated* O*-linked mannans at the cell wall surface [71], are as good as the wild type control cells to stimulate binding and cytokine production by human dendritic cells [98]. Moreover, there are some* C. albicans* strains whose immune sensing is independent of recognition via TLR4, suggesting that the fungus might be able to modulate the production of this cell wall component [99].

## 4. The GPI Anchors

**Figure 3:**
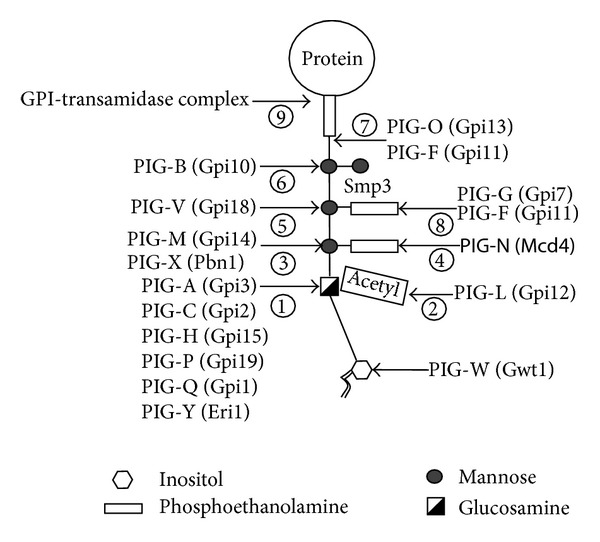
The GPI anchorage pathway. GPIs are glycolopids that act as a membrane anchor for many cell surface proteins and are composed of an inositol molecule that is sequentially modified in the ER with a Man3Glc*N* glycan and phosphoethanolamine groups. Numbers show sequential steps for the synthesis of a Man3Glc*N* glycan bearing GPI. A GPI-transamidase complex acts upon the phosphoethanolamine group linked to the terminal mannose to add the surface protein. The* C. albicans* Smp3 adds a fourth mannose residue that is essential for protein transfer; but in humans it is not essential for transamidation and its expression appears to be restricted to brain and colon.

The GPI anchors are complex structures that comprise a phospholipid tail, a glycan core, and a phosphoethanolamine linker (Figure 3). This structure is attached to the C-terminus of some eukaryotic proteins, allowing their anchoring to cell membranes or the wall. The core glycan, mannose(*α*1-2)mannose(*α*1-6)mannose(*α*1-4)glucosamine(*α*1-6)myo-inositol is highly conserved in eukaryotes, but it can be modified with other residues such as mannose, phosphoethanolamine (Etn-P), galactose, sialic acid, and others. The GPI synthetic pathway (Figure 3) initiates on the cytoplasmic side of the rER with the transfer of Glc*N*Ac from the UDP-Glc*N*Ac donor to phosphatidylinositol. This step requires several proteins that form complex (GPI-GnT), PIG-A/Gpi3, PIG-C/Gpi2, PIG-H/Gpi15, PIG-P/Gpi19, PIG-Q/Gpi1, and PIG-Y/Eri1 (mammals/yeast) [100–107]; see Table 4.

Next, Glc*N*Ac-PI is deacetylated by PIG-L/Gpi12, generating Glc*N*-PI [108], which requires crossing the rER membrane to continue the synthetic pathway within the lumen. This transport, as in the other protein modification described above, is carried out by a rER flippase. Then, inositol acylation takes place due to the acyltransferase activity of PIG-W/Gwt1, being the donor acyl-CoA [109, 110]. GPI mannosylation takes place using Dol-P-Man as mannose donor and begins with action of the mannosyltransferase PIG-M/Gpi14 in complex with PIG-X/Pbn1 that adds the first mannose (*α*1,4-linked) to Glc*N* [111]. The second (*α*1,6-linked) and third (*α*1,2-linked) mannose units are transferred by PIG-V/Gpi18 and PIGB/Gpi10 mannosyltransferases, respectively [112–115]. The enzyme Smp3 catalyses the addition of a fourth mannose residue (*α*1,2-linked) to Man-3 of the glycan core, being an essential step in yeast and Candida cells, as it is required for subsequent attachment of phosphoethanolamine [116, 117]. Mammalian cells mostly transfer trimannosyl-GPIs to proteins and do not require the addition of a fourth mannose residue, but a human ortholog of the yeast mannosyltransferase Smp3 that adds a fourth, *α*1,2-linked Man to trimannosyl GPI precursors has been identified, displaying high expression in brain and colon, suggesting that Man_4_-GPIs elaboration could be tissue-specific [117]. In* C. albicans*, Smp3 and is essential for viability and has been proposed to be a potential antifungal target [118].

An Etn-P unit can be attached to the first mannose of the glycan core as a side branch by PIG-N/Mcd4 and also to the second mannose by a complex of PIG-F/Gpi11 and PIG-G/Gpi7 [119–121]. Finally, a moiety of Etn-P is added to the third mannose of the core, being this residue the one bound to the protein through an amide link. A transferase association between PIG-O/Gpi13 and PIG-F/Gpi11 is responsible for this step [122, 123]. The GPI synthetic pathway is highly conserved, but GPIs can be further modified in the lipid and glycan moieties depending on genus, species, and protein type [124].

The GPI-anchored proteins have a C-terminal sequence that directs the attachment of a GPI anchor. The removal of the C-terminal GPI signal sequence and its replacement with GPI on the lumen of rER are catalyzed by the GPI transamidase (GPIT), which is a complex consisting of the membrane proteins PIG-K/Gpi8, GAA-1, PIG-S/Gpi-17, PIG-T/Gpi16, and PIG-U/Cdc91 [125, 126]. In the first step of the GPIT-catalyzed reaction, the GPI signal sequence is cleaved and the newly generated *α*-carbonyl group is attached via a thioester linkage to the PIG-K subunit of GPIT. Nucleophilic attack on the activated carbonyl by the amino group of the terminal EtN-P residue of GPI regenerates GPIT and yields a GPI-anchored protein.

After transfer, the inositol group introduced before mannosylation of the GPI precursor is removed in humans and yeast by the orthologous PGAP1/Bst1 deacylase ER proteins [127]. Yeast is able to remodel the shorter acyl chains of the diacylglycerol shortly after transfer to either base-labile C_26:0_/C_26:0_ diacylglycerols or to a base-stable ceramide consisting of C_18:0_ phytosphingosine and a hydroxy-C_26:0_ fatty acid. The remodeling initiates with the removal by PGAP3/Per1 of the acyl chain at the* sn*-2 position of the diacylglycerol [128, 129]. PGAP3-dependent removal of unsaturated fatty acyl chains at the* sn*-2 position occurs predominantly in the Golgi, whereas Per1 activity is located in the rER.

Next, a C_26:0_ acyl chain is introduced at* sn*-2 by the* O*-acyltransferase Gup1 that is the only enzyme involved in GPI anchor synthesis in* C. albicans* that has no human ortholog [130]. The mammalian PGAP2 protein is involved in the subsequent introduction of a saturated (C_18:0_) fatty acid at* sn*-2 [131]. Mutations in the yeast gene that encodes a homolog of PGAP2,* CWH43*, albeit a much larger protein, cause cell wall abnormalities consistent with defects in cell surface anchorage of GPI proteins. In mammals, remodeling at* sn*-2 requires prior inositol deacylation by PGAP1 [129]. The PGAP3- and PGAP2-dependent remodeling activities, in turn, are necessary for the GPI-anchored proteins to associate with lipid rafts.

### 4.1. Functions in Humans

The obvious role for GPI-anchors is the attachment of proteins to cell surface. Examples include cell surface receptors (e.g., folate receptor, CD14), cell adhesion molecules (e.g., neural cell adhesion molecule), cell surface hydrolases (e.g., alkaline phosphatase), and complement regulatory proteins (e.g., decay-accelerating factor [CD55]). Human diseases arise by failures in this posttranslational process, stressing its importance for proper function of human cells. The paroxysmal nocturnal hemoglobinuria is a consequence of lower surface expression of GPI-proteins, due to clonal acquired mutations in PIG-A [132]. Inherited mutation in the promoter region of PIG-M impairs the binding of the transcription factors, resulting in abrogation of GPI mannosylation, leading to propensity to venous thrombosis and seizures [133]. Other congenital diseases involving defective PIG anchoring have been recently described [134].

### 4.2. Functions in* C. albicans*

As in human cells, GPI synthesis is essential for* S. cerevisiae* growth [101, 135].* C. albicans* has 115 putative GPI-proteins, with diverse predicted functions, including adhesion to host tissues [136].* C. albicans GPI7* leads to an aberrant cell wall composition with increased chitin content and less protein abundance [137], while cells lacking Smp3 mannosyltransferase are nonviable [118]. Recently, it has been demonstrated that defects in GPI synthesis affect hypha growth [138]. Yadav et al. propose that Gpi2 and Gpi19 subunits of the GPI-GnT complex regulate ergosterol synthesis and RAS signaling, which explains the influence of GPI synthesis in the dimorphic switch [139]. Adhesins of the Als family are known to be GPI-proteins [140], so it is not surprising that virulence is attenuated in GPI mutants.

## 5. The Glycosylation Pathways as Potential Drug Targets against Fungal Infections

The information gathered in the last decades about the human glycosylation pathways has helped to differentiate the normal processes from those found in neoplastic cells, and these are now explored as potential strategies to treat cancer [141, 142]. Since protein glycosylation is a key process for* C. albicans* fitness and virulence attributes [4, 143], it is assumed that the development of inhibitors for any of the glycosylation pathways may assist in treatment of candidiasis. Tunicamycin is one of the oldest* N*-linked glycosylation inhibitors that has been thoroughly characterised over the last decades. It affects the elaboration of the* N*-linked glycan core [144], and tunicamycin-treated cells of* C. albicans* lose the viability [21] and the ability to generate biofilms [64], making this molecule a potential anti-*C. albicans* drug. However, the UDP-N-acetylglucosamine, dolichol phosphate GlcNAc-1-P transferase, the molecular target of this compound, is equally sensitive in both human and fungal cells [64].

A promising strategy for treatment of* C. albicans* infections could be found in the rER glucosidase inhibitors, which have been used in the experimental control of some viruses [145–149]. We have previously shown that* C. albicans* cells require processing of the* N*-linked glycan core in order to elongate their* N*-linked mannans, and loss of either glucosidase I or II led to virulence attenuation [21]. Since the human cells have a bypassing strategy for glucosidase trimming via the endomannosidase enzyme activity, it is feasible to conceive the potential low cytotoxicity of these drugs on the host cells. Celgosivir [150], PBDNJ0804-a deoxynojirimycin derivative [149], and CM-10-18 [147] are rER glucosidase I inhibitors that could show anti-*C. albicans* activity.

The* O*-linked mannosylation pathway can also be targeted for drug design. It was recently demonstrated that the rhodanine-3-acetic acid derivative OGT2468 is a PMT inhibitor in* S. cerevisiae* [151] and that it likely affects the same biosynthetic pathway in* C. albicans*. Whether this compound affects or not the elaboration of* O*-linked Man glycoprotein in human cells remains to be addressed.

The enzymes involved in GPI synthesis are also potential targets to develop new antifungal drugs. Gepinacin and E1210 were found to inhibit the fungal acyltransferase Gwt1, impairing the growth of fungal pathogens. Despite the functional similarity, gepinacin has no effect on the mammalian ortholog PIG-W. Assays on* C. albicans* cells treated with gepinacin indicate that they overexpose *β*-glucans on the wall surface, which triggers a better macrophage response [152–154]. Development of Smp3 or Gup1 inhibitors would be of value in view of their nonessential nature or absence in humans, respectively.

## 6. Conclusions

Metazoa (animals) and fungi derive from a common ancestor that existed ~1 billion years ago, nonetheless the basis of protein glycosylation pathways is strikingly conserved in spite of this period. In this review, we can look at the common bases and differences that emerge when comparing glycosylation mechanisms in* C. albicans* and humans.

The study of* C. albicans* glycosylation machinery is an important step to identify pharmacological targets to treat local or systemic candidiasis. Ideal pharmacological targets are represented by those elements only present in* Candida*. In the* N*-linked glycosylation pathway at least 16 GTs participate in mannan synthesis and are not present in humans (see Table 2), making this pathway an attractive alternative for drug design. Thus far, some promising approaches have been done with glucosidase inhibitors, but their toxicity in human cells remains to be addressed. In addition, rER-mannosidase inhibitors could be used as an alternative approach, as fungal cells only contain one mannosidase class I within the rER, and its loss is associated with virulence attenuation [21].

The above data indicate that fungal glycosylation pathways are promising for inhibitory compound screening that are species specific, both because of the presence of many nonhomologous proteins identified in* C. albicans*, particularly in the* N*-glycosylation pathway, and also because of the presence of homologous proteins that have a low degree of identity. Further studies should focus on developing compounds to inhibit the essential functions of glycosylation pathways taking into account these facts.

## Figures and Tables

**Table 1:** Human and *C. albicans* homolog proteins involved in the *N*-linked glycosylation pathway.

Protein function	Protein Human/*Candida *	Human ID∗	*Candida* ID∗	Id∗∗	COV∗∗∗
*Glycosyltransferases *					
UDP-GlcNAc: Dol-P N-acetylglucosaminephosphotransferase	GT1/Alg7	NP_001373.2	XP_716028.1	37%	87%
UDP-GlcNAc: Dol-PP-GlcNAc N-acetylglucosaminyl transferase	Alg13	NP_001093392.1	XP_718987.1	30%	60%
UDP-GlcNAc: Dol-PP-GlcNAc N-acetylglucosaminyl transferase	Alg14	NP_659425.1	XP_717086.1	37%	77%
GPD-Man: Dol-PP-GlcNAc_2_ *β*1,4-mannosyltransferase	Alg1	NP_061982.3	XP_711858.1	34%	88%
GPD-Man: Dol-PP-GlcNAc_2_Man *α*1,3-*α*1,6-mannosyltransferase	Alg2	NP_149078.1	XP_710581.1	41%	96%
GPD-Man: Dol-PP-GlcNAc_2_Man_3_ and Man_4_ *α*1,2-mannosyltransferase	Alg11	NP_001004127.2	XP_712508.1	36%	67%
Dol-P-Man: Dol-PP-GlcNAc_2_Man_5_ *α*1,3-mannosyltransferase	Alg3	NP_001006942.1	XP_712080.1	30%	81%
Dol-P-Man: Dol-PP-GlcNAc_2_Man_7_ *α*1,6-mannosyltransferase	Alg12	NP_077010.1	XP_716986.1	33%	86%
Dol-PP-Man: Dol-PP-GlcNAc_2_Man_6_ and Man8 *α*1,3-mannosyltransferase	Alg9	NP_001071158.1	XP_713886.1	36%	93%
Dol-PP-Glc: Dol-PP-GlcNAc_2_Man_9_ *α*1,3-glucosyltransferase	Alg6	NP_037471.2	XP_711029.1	37%	87%
Dol-PP-Glc: Dol-PP-GlcNAc_2_Man_9_Glc *α*1,3-glucosyltransferase	Alg8	NP_001007028.1	XP_721736.1	41%	80%
Dol-PP-Glc: Dol-PP-GlcNAc_2_Man_9_Glc_2_ *α*1,2-glucosyltransferase	Alg10	NP_116223.3	XP_714677.1	32%	96%
*Post flipping chaperone *	Rft1	NP_443091.1	XP_717469.1	27%	85%
*OST components *	RBPH1/Ost1	NP_002941.1	XP_714694.1	27%	85%
	DAD1/Ost2	NP_001335.1	XP_714366.1	40%	81%
	N33/Ost3	NP_006756.2	XP_721902.1	23%	77%
	Ost4	NP_001128165.1	EEQ47486.1	39%	51%
	IAP/Ost6	NP_115497.4	XP_716090.1	22%	84%
	RIBIIR/SwpI	NP_002942.2	XP_721287.1	26%	58%
	OST48 /Wbp1	NP_005207.2	XP_713903.1	24%	91%
	STT3A/Stt3	NP_001265432.1	XP_722527.1	56%	94%
	STT3B	NP_849193.1		57%	
*Folding Sensor *	UGGT1/Kre5	NP_064505.1	XP_719987.1	60%	57%
*Glycosidases *					
Glucosidase I	MOGS/Cwh41	NP_001139630.1	ABB97046.1	29%	91%
Glucosidase II	GANAB/Rot2	NP_938148.1	XP_716812.1	38%	98%
	GLU2B/Gtb1	NP_002734.2	XP_717976.1	39%	57%
ER *α*1,2-mannosidase I	MAN1B1/MnsI	NP_057303.2	XP_713641.1	45%	77%

*Accession number at NCBI database.

**Identity and ***coverage from BLAST alignment between human and *C. albicans* homolog sequences, respectively.

**Table 2:** *C. albicans* nonhomologous proteins involved in the *N*-linked, *O*-linked, and GPI-anchor pathways.

Protein function	Protein	ID∗
*N-glycosylation *		
Golgi *α*1,6-mannosyltransferase	Och1	XP_716632
Golgi *α*1,6-mannosyltransferase complex M-Pol I	Mnn9	XP_716624.1
	Van1	XP_719719.1
Golgi *α*1,6-mannosyltransferase complex M-Pol II	Mnn9	XP_716624.1
	Anp1	XP_714464.1
	Mnn10	XP_713339.1
	Mnn11	XP_721427.1
	Hoc1	XP_716693.1
Golgi *α*1,2-mannosyltransferases	Mnn5	XP_713952.1
	Mnt4	XP_711944.1
	Mnt5	XP_712920.1
Golgi *α*1,3-mannosyltransferases	Mnn1	XP_720587.1
Golgi *β*1,2-mannosyltransferases	Bmt1	XP_719878.1
	Bmt2	XP_710865.1
	Bmt3	XP_717972.1
	Bmt4	XP_719173.1
Golgi phosphomannosyltransferase	Mnt3	XP_710267.1
	Mnt5	XP_713952.1
*O-glycosylation *		
Protein *O*-mannosyltransferase	Pmt1	XP_716926.1
Protein *O*-mannosyltransferase	Pmt5	XP_719311.1
Protein* O*-mannosyltransferase	Pmt6	XP_717283.1
Golgi *α*1,2-mannosyltransferases	Mnt1	XP_721742.1
Golgi *α*1,2-mannosyltransferases	Mnt2	XP_721740.1
*GPI anchor *		
*α*1,2-mannosyltransferase (brain and colon)	Smp3	XP_715268.1
*O*-acyltransferase	Gup1	XP_722305.1

*Accession number at NCBI database.

**Table 3:** Human and *C. albicans* homolog proteins involved in the *O-*mannosylation pathway.

Protein function	Protein H/Ca	Human ID∗	Candida ID∗	Id∗∗	Cov∗∗∗
Protein* O*-mannosyltransferase	POMT2/Pmt2	NP_037514.2	XP_719907.1	36%	89%
Protein *O*-mannosyltransferase	POMT1/Pmt4	NP_001129586.1	XP_714280.1	34%	80%

*Accession number at NCBI database.

**Identity and ***coverage from BLAST alignment between human and *C. albicans* homolog sequences, respectively.

**Table 4:** Human and *C. albicans* homologous proteins involved in GPI glycosylation.

Protein function	Protein H/Ca	Human ID∗	*Candida* ID∗	Id∗∗	Cov∗∗∗
GlcNAc-PI synthesis	PIG-A/Gpi3	NP_002632.1	XP_717439.1	49%	94%
	PIG-C/Gpi2	NP_714969.1	XP_717493.1	31%	90%
	PIG-H/Gpi15	NP_004560.1	XP_718197.1	33%	44%
	PIG-P/Gpi19	NP_710149.1	XP_714916.1	33%	37%
	PIG-Q/Gpi1	NP_683721.1	XP_714683.1	30%	42%
	PIG-Y/Eri1	NP_001036081.1	XP_715355.1	64%	9%
GlcNAc-PI de-N-acetylation	PIG-L/Gpi12	NP_004269.1	XP_723585.1	29%	55%
Inositol acylation	PIG-W/Gwt1	NP_848612.2	XP_712842.1	28%	99%
*α*1,6-mannosyltransferase	PIG-M/Gpi14	NP_660150.1	XP_722653.1	38%	94%
*α*1,6-mannosyltransferase	PIG-X/Pbn1	NP_001159776.1	XP_716695.1	19%	24%
Etn-P transfer to Man-1	PIG-N/Mcd4	NP_789744.1	XP_716313.1	37%	95%
*α*1,6-mannosyltransferase	PIG-V/Gpi18	NP_060307.2	XP_713712.1	24%	82%
*α*1,2-mannosyltransferase	PIG-B/Gpi10	NP_004846.4	XP_721904.1	31%	97%
Etn-P transfer to Man-3	PIG-O/Gpi13	NP_001188413.1	XP_720956.1	38%	53%
Etn-P transfer to Man-2 and 3	PIG-F/Gpi11	NP_002634.1	XP_720511	35%	44%
Etn-P transfer to Man-2	PIG-G/Gpi7	NP_001120650.1	XP_710743.1	38%	47%
*α*1,2-mannosyltransferase	Smp3	NP_079439.2	XP_715333.1	26%	91%
Etn-P transfer to Man-2	Gpi7	NP_001120650.1	EEQ42670.1	40%	72%
**GPI transamidase**	PIG-K/Gpi8	NP_005473.1	XP_711741.1	56%	78%
	GAA1	NP_003792.1	XP_710522.1	35%	49%
	PIG-S/Gpi17	NP_149975.1	XP_716135.1	27%	48%
	PIG-T/Gpi16	NP_057021.2	XP_720200.1	29%	94%
	PIG-U/Cdc91	NP_536724.1	XP_720773.1	24%	92%
Inositol deacylation	PGAP-1/Bst1	NP_079265.2	XP_713657.1	31%	75%
*sn-*2 deacylation	PGAP3/Per1	NP_219487.3	XP_712020.1	26%	95%
*sn-*2 acylation	PGAP2/Cwh43	NP_001269969.1	XP_717850.1		

*Accession number at NCBI database.

**Identity and ***coverage from BLAST alignment between human and *C. albicans* homologous sequences, respectively.
